# Tuberculosis associated with *Mycobacterium tuberculosis *Beijing and non-Beijing genotypes: a clinical and immunological comparison

**DOI:** 10.1186/1471-2334-6-105

**Published:** 2006-07-05

**Authors:** Yong-Jiang Sun, TK Lim, Adrian Kheng Yeow Ong, Benjamin Choon Heng Ho, Geok Teng Seah, Nicholas I Paton

**Affiliations:** 1 Department of Infectious Diseases, Tan Tock Seng Hospital, Republic of Singapore; 2 Department of Medicine, National University of Singapore, Republic of Singapore; 3 Department of General Medicine, Tan Tock Seng Hospital, Republic of Singapore; 4 Department of Microbiology, National University of Singapore, Republic of Singapore

## Abstract

**Background:**

The *Mycobacterium tuberculosis *Beijing genotype is biologically different from other genotypes. We aimed to clinically and immunologically compare human tuberculosis caused by Beijing and non-Beijing strains.

**Methods:**

Pulmonary tuberculosis patients were prospectively enrolled and grouped by their *M. tuberculosis *genotypes. The clinical features, plasma cytokine levels, and cytokine gene expression levels in peripheral blood mononuclear cells (PBMC) were compared between the patients in Beijing and non-Beijing groups.

**Results:**

Patients in the Beijing group were characterized by significantly lower frequency of fever (odds ratio, 0.12, *p *= 0.008) and pulmonary cavitation (odds ratio, 0.2, *p *= 0.049). Night sweats were also significantly less frequent by univariate analysis, and the duration of cough prior to diagnosis was longer in Beijing compared to non-Beijing groups (medians, 60 versus 30 days, *p *= 0.048). The plasma and gene expression levels of interferon (IFN) γ and interleukin (IL)-18 were similar in the two groups. However, patients in the non-Beijing group had significantly increased IL-4 gene expression (*p *= 0.018) and lower IFN-γ : IL-4 cDNA copy number ratios (*p *= 0.01).

**Conclusion:**

Patients with tuberculosis caused by Beijing strains appear to be less symptomatic than those who have disease caused by other strains. Th1 immune responses are similar in patients infected with Beijing and non-Beijing strains, but non-Beijing strains activate more Th2 immune responses compared with Beijing strains, as evidenced by increased IL-4 expression.

## Background

The *Mycobacterium tuberculosis *Beijing genotype was first described in 1995 because of its predominance in the Beijing area of China, the high similarity of IS*6110 *RFLP patterns, and the identical polymorphism pattern of the direct repeat region of *M. tuberculosis *genome between isolates [1]. Thereafter, it has been shown that strains of this genotype are also predominant in other East Asian areas [2-4] and prevalent all over the world [5,6]. The selective advantage of the Beijing genotype has led to the postulation that it may be an 'escape variant' of mass bacillus Calmette-Guérin (BCG) vaccination [1]. In addition, Beijing strains have been reported to be associated with various phenotypes, such as drug-resistance [2,7-10], treatment failure and tuberculosis relapse [11], and febrile response to treatment [12]. Studies in mice have shown that Beijing strains are more virulent and do not favor Th1 immune response [13,14].

These findings suggest that the Beijing genotype strains behave differently from non-Beijing genotype strains. However, there has been no study performed to compare the immune responses in patients infected by Beijing and non-Beijing strains. In this prospective study, we aimed to determine whether there are clinical and biological differences in the host response to infections caused by Beijing and non-Beijing strains. In particular we hypothesized that Beijing strains elicit a weaker Th1 immune response, as shown by a reduced production of plasma IFN-γ.

## Methods

### Subjects and setting

Inpatients with pulmonary tuberculosis were prospectively and consecutively enrolled from Tan Tock Seng Hospital (TTSH), and National University Hospital (NUH) from September 2004 to May 2005. Subject inclusion criteria were: (1) clinical presentation and radiographic findings considered to be strongly suggestive of pulmonary tuberculosis by the primary physicians, (2) ≥ 18 years old, (3) antituberculous treatment naïve or not longer than 48 hours, (4) able and willing to give informed consent. Patients who had a past history of tuberculosis, who were known to have human immunodeficiency virus (HIV) infection or other coexisting systemic diseases, such as chronic renal failure, diabetes mellitus, and neoplastic diseases, and pregnant or breastfeeding women were excluded. Approval for this study was granted by the Ethics Committee of the National Healthcare Group (NHG), Singapore. Written informed consent was obtained from all study subjects.

### Demographic and clinical data collection

Demographic (date of birth, place of birth, sex, and race), clinical, and routine laboratory testing data were collected by interview of patients and/or from medical records. The data of BCG scar, BCG vaccination history, TB contact history, and risk factors for HIV infection were collected by interview of the subjects. The information on clinical symptoms and their durations, including cough, fever, night sweats, haemoptysis, weight loss, appetite loss, and chest pain, were first collected from medical records and confirmed through patient interviews. Routine temperature charts were examined covering the time of admission until the time of enrolment. Axillary temperature was recorded every 6 hours using a digital thermometer in both hospitals. The patient was defined having fever if there was a recorded temperature of > 37.5°C. Routine laboratory test data including total and differential white blood cell counts, hemoglobin, sputum smear, culture and drug-susceptibility test results were collected. Anemia was defined when hemoglobin < 13 g/dL in males and < 11.8 g/dL in females. All patients were screened for risk factors of HIV infection and offered HIV serology testing.

Chest X-ray (CXR) films were reviewed at each institution by a single clinician (TKL and AKYO) experienced in the diagnosis and management of tuberculosis who were blinded to *M. tuberculosis *genotyping results. The disease extent on CXR was classified as: (1) unilateral disease, (2) bilateral disease, (3) cavitary disease, (4) pleural effusion, and (5) miliary disease.

### DNA extraction and genotyping of *M. tuberculosis*

Sputum specimens were collected from each subject at enrolment. Sputum samples were first decontaminated and liquefied with 1% N-Acetyl-L-Cysteine (NALC)-2% NaOH. DNA was extracted using phenol-chloroform/isoamyl alcohol, precipitated with absolute ethanol and washed with 70% ethanol according to standard protocol. DNA was finally dissolved in 50 μl distilled water.

Spoligotyping was performed using a commercial kit (Isogen Bioscience BV, Maarssen, the Netherlands) according to the manufacturer's protocol. Spoligotyping patterns were translated to 15 octal codes as suggested before [15]. Mycobacterial interspersed repetitive unit-variable number tandem repeat (MIRU-VNTR) typing was performed as previously described [16,17]. Two μl of sputum DNA sample was used in each PCR reaction for these genotyping analyses. MIRU-VNTR allele number was manually converted according to amplicon size.

### Isolation of plasma and PBMC

Blood samples collected in heparinized tubes were centrifuged at 300 × g for 10 minutes to separate plasma and blood cells. Plasma was recovered and further centrifuged at 14,000 rpm for 15 minutes at 4°C, and then recovered in 0.5 ml aliquots and stored in -80°C freezer. The precipitated blood cells were resuspended in appropriate volume of RPMI 1640 Media and subjected to PBMC isolation by Ficoll-Paque gradient centrifugation.

### Cytokine ELISA

Blood plasma levels of IFN-γ, IL-2, IL-4, IL-13, and IL-18 were measured using quantitative ELISA kits (Bender MedSystems, Austria) according to the manufacturer's protocols and each sample was assayed in duplicate. The sensitivity of these kits were 0.66 pg/ml, 2.3 pg/ml, 0.66 pg/ml, 0.99 pg/ml, 9.2 pg/ml, respectively. A high sensitivity (0.06 pg/ml) IFN-γ kit was used to measure some samples with very low level of IFN-γ.

### Quantification of cDNA by Real-Time PCR

Total RNA was extracted from 2 × 10^6 ^peripheral blood mononuclear cells (PBMCs) using TRIZOL^® ^reagent (Invitrogen, California, USA) according to the manufacturer's protocol. cDNA was reverse transcribed from total RNA using the 1st Strand cDNA Synthesis Kit for RT-PCR (AMV) (Roche Applied Science). The cDNA of reverse transcribed cytokine genes IFN-γ, IL-2, IL-4, IL-13, and IL-18, and housekeeping gene β-actin were quantified by quantitative real-time PCR using the LightCycler™ FastStart Master Sybr^®^Green I kit on the Roche LightCycler apparatus (Roche Applied Science). The quantitative real-time PCR primers and standards for these genes were obtained from Search-LC GmbH (Heidelberg, Germany) and the manufacturer's protocols were followed. Melting curve analysis was performed to confirm the identity and specificity of PCR products. All cytokine cDNA copies were normalized to the housekeeping gene β-actin.

### Statistical analysis

χ^2 ^test or Fisher's exact test was used to analyze categorical variables. Student's *t *test was used to compare normally distributed continuous variables and those variables which were normally distributed after log-transformation. Multivariate logistic regression was used to adjust for potential confounding factors. A *p *value of <0.05 was considered statistically significant. Odds ratios (OR) and 95% confidence intervals (CI) were calculated.

## Results

### Patient enrolment and determination of *M. tuberculosis *genotypes

A total of 54 patients were enrolled in this study (35 from TTSH and 19 from NUH). One of the 54 patients was found to be HIV-infected, thus was excluded from further analysis. Of the remaining patients, 16 tested negative for HIV antibodies. Those who were not tested did not have any apparent risk factors for HIV infection nor any evidence of immune suppression noted on physical examination. Two further patients were excluded because they were unable to provide a sputum specimen for genotyping. Nine patients were excluded because they were culture negative, sputum smear negative for acid-fast bacilli and did not have *M. tuberculosis *detected by either spoligotyping or MIRU-VNTR typing.

Forty-two patients had culture-proven, drug-susceptible (to rifampicin, streptomycin, isoniazid, and ethambutol) pulmonary tuberculosis. Of these 42, 38 (90.5%) were smear positive. *M. tuberculosis *DNA was detected in the sputum sample and successfully typed by MIRU-VNTR typing in 41 cases, and by spoligotyping in 39 cases. All the cases of failure of genotyping were also smear negative. The single case that could not be typed by either method was excluded from further analysis. Therefore, the study analysis concerns 41 culture-proven tuberculosis patients with genotyping data.

Twenty-one (51.2%, 21/41) patients were infected with Beijing strains (Table 1). Of these, 19 isolates had the typical Beijing spoligotype which is absent of spacers 1–34 but contains spacers 35–43 [18,19]. One isolate (N04) showed Beijing-like spoligotype which has been demonstrated also belonging to the Beijing lineage [19]. The remaining one isolate (N07) was not detected by spoligotyping, but MIRU-VNTR typing showed that it had a typical cluster MIRU-VNTR pattern (223325173533) specific for Beijing strains. Based on spoligotypes and/or MIRU-VNTR patterns, the remaining 20 isolates were assigned to non-Beijing genotypes: the Haarlem family (6 isolates, 14.6%), the Latin American and Mediterranean (LAM) family (1 isolate, 2.4%), the T family (4 isolates, 9.8%), and the East Africa Indian (EAI) family (9 isolates, 22%).

**Table 1:** Genotyping result of *M. tuberculosis *isolates

Isolates	MIRU-VNTR	Spoligotypes	Genotypes
N07	223325173533	ND	Beijing
N08	223325173533	000000000003771	Beijing
T21	223325173533	000000000003771	Beijing
T32	223325173533	000000000003771	Beijing
T38	223325173533	000000000003771	Beijing
T41	223325173533	000000000003771	Beijing
T44	223325173533	000000000003771	Beijing
T45	223325173533	000000000003771	Beijing
T47	223325173533	000000000003771	Beijing
T49	223325173533	000000000003771	Beijing
T55	223325173533	000000000003771	Beijing
N14	223325173333	000000000003771	Beijing
T51	223325173433	000000000003771	Beijing
T18	223325193533	000000000003771	Beijing
N05	223325153513	000000000003771	Beijing
T53	223325163534	000000000003771	Beijing
N13	222325173543	000000000003771	Beijing
T11	222325173543	000000000003771	Beijing
T33	223325171531	000000000003771	Beijing
T52	233225173443	000000000003771	Beijing
N04	222325173543	000000000000771	Beijing
T34^a^	2223251735n3	400000001000200	Haarlem
T03	222325143323	557367770000661	Haarlem
N26	222325153323	777340770720771	Haarlem
T50	226325153323	477777774020771	Haarlem
T12	122325153423	776001370020771	Haarlem
T19	222425153324	777777777720771	Haarlem
N22	224326143327	777777606760771	LAM
T37	225125113322	777777776760600	T
T36	224226123323	777377236760771	T
N28	242325152312	777367770760621	T
T42	232325142322	767777777760771	T
T24	255326222513	577377777413771	EAI
T35	254326223432	677377477413771	EAI
T30	274326223432	677377477413771	EAI
T54	254326223432	677777477413771	EAI
N09	254225223522	477777477413771	EAI
T02	254326223434	477777777413071	EAI
T40	254326223432	000000007413771	EAI
T48	364225223533	777777000000011	EAI
N01	264326223432	ND	EAI

MIRU-VNTR patterns are based on the 12 loci as described in reference [16]. Spoligotypes are presented as 15 octal codes as recommended in reference [15]. ND, not detected.

^a^n = 10.

### Demographic and epidemiological characteristics

Table 2 presents the demographic and epidemiologic characteristics of the subjects segregated by *M. tuberculosis *Beijing and non-Beijing genotypes. The distributions of sex, ethnicity, and age did not differ significantly between the two groups. The frequency of past BCG vaccination and the proportion of smear positive cases were similar in the two groups. The median score for the positive smears was 3+ in each group. Four patients had known contact history with family members (2 in each group). The distributions of Beijing and non-Beijing isolates were similar at the two hospitals.

**Table 2:** Characteristics of patients by *M. tuberculosis *genotypes

Characteristics	Beijing n = 21 (%)	Non-Beijing n = 20 (%)	p Value
Male sex	14 (66.7)	10 (50.0)	0.297
Ethnicity			0.286
Chinese	14 (66.7)	12 (60.0)	
Malay	7 (33.3)	6 (30.0)	
Indian	0 (0)	2 (10.0)	
Age (year)			0.325
≤ 30	4 (19.0)	6 (30.0)	
31–59	12 (57.1)	11 (55.0)	
≥ 60	5 (23.8)	3 (15.0)	
BCG			0.303
Yes	11 (52.4)	13 (65.0)	
No/unknown	10 (47.6)	7 (35.0)	
Sputum Smear			0.486
Positive	19 (90.5)	19 (95.0)	
Median scores	3+	3+	
Known contact history	2 (9.5)	2 (10.0)	0.959
Residentships			0.141
Singapore resident^a^	17 (80.9)	12 (60.0)	
Foreigner	4 (19.1)	8 (40.0)	
Hospitals			0.796
TTSH	15 (71.4)	15 (75.0)	
NUH	6 (28.6)	5 (25.0)	

**NOTE**. Beijing, Beijing genotype; non-Beijing, non-Beijing genotypes; BCG, bacillus Calmette-Guérin.

^a^Citizens and permanent residents.

### Clinical and radiological features

As shown in Table 3, cough was the most common symptom for the pulmonary tuberculosis patients, 38 of the 41 (92.7%) patients had cough. The less frequent manifestations were fever (26/41, 63.4%), and weight loss (28/41 68.3%). The frequencies of haemoptysis, appetite loss, night sweats, and chest pain were even lower.

**Table 3:** Clinical and chest X-ray manifestations of patients by *M. Tuberculosis *genotypes

Parameters	Beijing n = 21 (%)	Non-Beijing n = 20 (%)	Univariate analysis	
			
			OR (95% CI)	p value
Clinical presentations				
Cough	18 (85.7)	20 (100)	0.00 (0.00–1.27)	0.125
Median duration (range)^a^	60 (14–1440)	30 (2–180)		0.048
Fever	9 (42.9)	17 (85.0)	0.13 (0.03–0.57)	0.005
Haemoptysis	4 (19.0)	6 (30.0)	0.55 (0.14–2.22)	0.414
Weight loss	16 (76.2)	12 (60.0)	2.13 (0.58–7.87)	0.265
Appetite loss	6 (28.6)	4 (20.0)	1.60 (0.40–6.39)	0.523
Night sweats	2 (9.5)	7 (35.0)	0.20 (0.04–0.99)	0.049
Chest pain	2 (9.5)	4 (20.0)	0.42 (0.08–2.29)	0.343
CXR presentations				
Unilateral disease	7 (33.3)	8 (40.0)	0.75 (0.22–2.61)	0.658
Bilateral disease	13 (61.9)	11 (55.0)	1.33 (0.39–4.52)	0.654
Pleural effusion	1 (4.8)	1 (5.0)	0.95 (0.09–9.75)	0.972
Cavity	2 (9.5)	7 (35.0)	0.20 (0.04–0.99)	0.049
WBC count (cells/μl)				
Total WBC (mean ± SD)	8629 ± 3331	10415 ± 3996		0.150
Lymphocytes (mean ± SD)	1376 ± 614	1563 ± 983		0.496
Monocytes (mean ± SD)	656 ± 340	901 ± 361		0.014
Eosinophils (mean ± SD)	144 ± 194	80 ± 82		0.193
Basophils (mean ± SD)	32 ± 17	40 ± 43		0.474
Anemia^b^	14 (66.7)	13 (65.0)	1.08 (0.31–3.81)	0.910
Hemoglobin (g/dL)				
Male (mean ± SD)	11.8 ± 1.2	11.3 ± 1.6		
Female (mean ± SD)	11.6 ± 1.9	11.3 ± 1.4		

Beijing, Beijing genotype; non-Beijing, non-Beijing genotypes; OR, odds ratio; CI, confidence interval; CXR, chest X-ray; WBC, white blood cells; SD, standard deviation.

^a^Unit is in day.

^b^Hemoglobin < 13 g/dL in males and < 11.8 g/dL in females.

**Figure 1:**
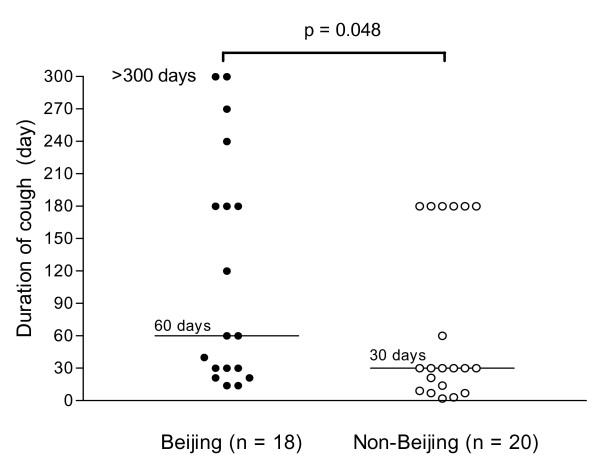
**Cough duration (day) of patients infected with Beijing and non-Beijing strains**. Cough durations of the pulmonary tuberculosis patients before diagnosis were compared by Beijing and non-Beijing groups. Patients infected with Beijing strains had significantly longer cough (median, 60 days) compared to those infected with non-Beijing strains (median, 30 days). Two patients in the Beijing group who had 4-year long cough before the diagnosis are expressed as > 300 days. The two groups differed significantly in the duration of cough (*p *= 0.048).

There was no significant difference in the frequency of cough between Beijing and non-Beijing infected patients (*p *= 0.125). However, in the 18 patients who had cough and were infected with Beijing strains, the duration of cough before the diagnosis varied widely from 14 to 1440 days (4 years) with a median of 60 days. Only 39% (7/18) of such patients were identified and diagnosed within one month. For the patients infected with non-Beijing strains, the range of the duration was much narrower, from 2 to 180 days with a median of 30 days. Much higher proportion (65%, 13/20) of the patients was identified within one month. The individual cough duration is shown in Figure 1. The difference between the two groups is statistically significant (*p *= 0.048), indicating that patients infected with Beijing strains presented with longer duration of cough before the diagnosis.

Fever was recorded in 9 (42.9%) of the 21 patients infected with Beijing strains and 17 (85.0%) of the 20 patients infected with non-Beijing strains [OR (95% CI), 0.13 (0.03–0.57), *p *= 0.005]. In addition to fever, night sweats were less frequently reported in patients infected with Beijing strains [OR (95% CI), 0.20 (0.04–0.99), *p *= 0.049]. To further test these associations, we performed multivariate logistic regression analysis. Independent variables included sex, age, and ethnicity, though they did not differ significantly between the two groups, as well as fever and night sweats. The inverse association between febrile response and Beijing strains remained strong after multivariate adjustment [OR (95% CI), 0.12 (0.02–0.63), *p *= 0.008]. However, the inverse association between night sweats and Beijing strains was no longer statistically significant after the multivariate adjustment [OR (95% CI), 0.20 (0.02–1.75), *p *= 0.145].

The chest radiographic abnormalities associated with infections of Beijing and non-Beijing strains were compared (Table 3). There was significantly less cavitary disease in patients infected with Beijing strains [OR (95% CI), 0.20 (0.04–0.99), *p *= 0.049]. No difference was observed in the frequency of unilateral and bilateral non-cavitary disease, and pleural effusion. Miliary disease was not found in the subjects.

The frequency of anemia in the entire study group was 65.9% (27/41), and there was no difference between the two groups (Table 3). Table 3 also presents total and differential counts of white blood cells of the patients. The mean counts for total white blood cells, lymphocytes, eosinophils, and basophils were not significant different in each group. The mean monocyte count was significantly higher in the non-Beijing group (mean, 901/μl) compared to the Beijing group (mean, 656/μl) (*p *= 0.014).

### Plasma cytokine and cytokine gene expression analysis

**Figure 2:**
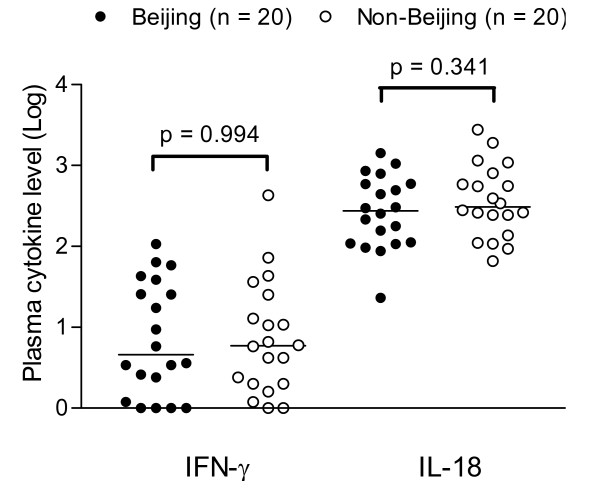
**Plasma cytokine levels**. Plasma levels of IFN-γ and IL-18 in pulmonary tuberculosis patients were quantified using ELISA assay. Patients infected with Beijing and non-Beijing strains showed similar levels of IFN-γ (*p *= 0.994) and IL-18 level (*p *= 0.341). The bars indicate the log median levels in each group.

Blood samples were collected from 40 of the 41 tuberculosis patients. Plasma IL-2, IL-4, and IL-13 were not detectable by the ELISA kits, thus not shown. The plasma levels of IFN-γ and IL-18 were similar between patients infected with Beijing and non-Beijing strains (Figure 2)

Of the 40 blood samples collected and prepared for cytokine gene expression analysis, the first 15 samples had very low RNA yields, thus the cDNA transcribed from these samples was too low to be reliably quantified. The results presented below pertain to the 25 samples in which satisfactory RNA samples were obtained. The 25 samples were from 12 patients infected with Beijing strains (8 males, 66.7%; mean age, 52, SD, 14) and 13 patients infected with non-Beijing strains (5 males, 38.5%; mean age, 48, SD, 21). These characteristics were similar between the subgroup patients and the whole group of study subjects (refer to Table 2).

**Figure 3:**
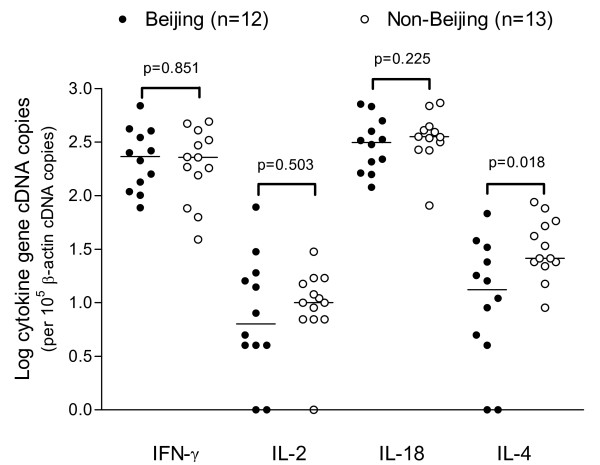
**Comparison of cDNA copies of cytokine genes**. cDNA copies of IFN-γ, IL-2, IL-18, and IL-4 were compared by Beijing and non-Beijing groups. The two groups of patients showed no difference in the expression of IFN-γ, IL-2, and IL-18 genes (*p *> 0.05). But patients infected with non-Beijing strains had significantly higher expressed cDNA copies of IL-4 than those with Beijing strains (*p *= 0.018). Log medians of cDNA copies are indicated by bars.

The expression of cytokine genes was standardized as per 10^5 ^β-actin cDNA copies. The expression level of IL-13 was too low to be detected by the quantitative real-time PCR, thus not shown. As shown in Figure 3, the cDNA copies of IFN-γ, IL-2 and IL-18 did not differ significantly between the Beijing and non-Beijing groups. The median cDNA copies for IFN-γ, IL-2 and IL-18 were respectively 232 (range, 77–691), 7 (range, 0–78), and 314 (range, 120–715) in patients infected with Beijing strains, and 228 (range, 39–491), 10 (range, 0–30), 355 (range, 81–1067) in patients infected with non-Beijing strains. The cDNA copies of IL-4 were significantly higher in patients infected with non-Beijing strains (median = 26, range, 9–87) compared to those with Beijing strains (median = 16, range, 0–68) (*p *= 0.018).

**Figure 4:**
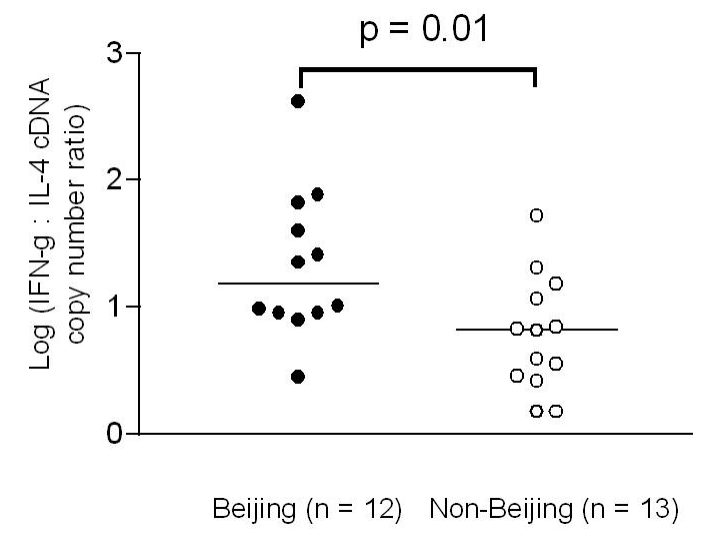
**IFN-γ : IL-4 cDNA copy number ratio of pulmonary tuberculosis patients**. Patients infected with non-Beijing strains had significantly lower ratios than those infected with Beijing strains (*p *= 0.01). The bars indicate log median ratios.

We also compared the IFN-γ : IL-4 cDNA copy number ratio between the Beijing and non-Beijing groups. Two patients who had no detectable IL-4 cDNA copies were assumed having 1 cDNA copy in order to calculate the ratios. Patients infected with non-Beijing strains showed significantly lower IFN-γ : IL-4 cDNA copy number ratios than those infected with Beijing strains (*p *= 0.01) (Figure 4).

## Discussion

In this study we recruited consecutive patients with clinically defined pulmonary tuberculosis to analyze the clinical and immunological differences between *M. tuberculosis *genotypes. All the subjects had microbiologically confirmed tuberculosis, and the manner of recruitment and pattern of symptoms suggests that they are likely to be representative of the population of tuberculosis patients presenting to a typical general practitioner, polyclinic or hospital emergency department in Singapore. In spite of the reluctance of some patients to undergo HIV testing, we were able to definitively exclude HIV co-infection in many of the participants, and establish the absence of risk factors in the remainder. Given that the rate of HIV coinfection in tuberculosis patients is less than 1% in Singapore (unpublished data), it is unlikely that a significant proportion of the remainder would be HIV positive.

Fever is a common manifestation of pulmonary tuberculosis and is one of the few that can be measured objectively. The frequency with which fever has been observed in patients with tuberculosis varies widely from approximately 34% to 94% [20-23]. Patients with fever are more often symptomatic, more likely to have advanced disease, more likely to be smear positive, and tend to be younger [21,22]. In this study we made the interesting and potentially important observation that 85% of patients infected with non-Beijing strains had fever, whereas only 43% of those infected with Beijing strains had fever, and this large difference is statistically significant.

We used a definition of fever that depended on objective observations made while patients were hospitalized, and recorded by nursing staff who were obviously unaware of the genotype of *M. tuberculosis *with which the patient is infected. In addition to fever, we also found that night sweats were significantly less common in patients infected with Beijing strains. This latter finding agrees with the findings of a large study performed in Russia by Drobniewski et al. [24] to examine differences between patients infected with Beijing and non-Beijing strains. In that study, night sweats were found significantly less often (OR, 0.7) in patients infected with Beijing strains and the association remained significant after multivariate adjustment. However, in that study there was no significant difference in the frequency of fever between Beijing and non-Beijing groups. The definition of fever was not given and the overall rate (approximately 30% in both groups) suggests that some under-reporting of fever may have occurred. Although we found that the frequency of fever and of night sweats differed between the Beijing and non-Beijing groups, after multivariate analysis only fever remained significant. This is not surprising as the two symptoms often occur together in the same patient, and so it is difficult to ascertain an independent effect. The important point is that our study agrees with the study from Russia that there is a difference in the clinical systemic host response to Beijing and non-Beijing genotype strains. In addition, Drobniewski et al. [24] have suggested that sub-Beijing families may be different in pathogenicity or transmissibility. The Beijing isolates in our study and those in the Russian study may belong to different sub-Beijing families and this may be related with the inconsistence between the two studies.

The presence of a constitutional symptom such as fever will usually lead patients to seek medical attention. Conversely, the absence of such symptoms or a more indolent presentation may therefore lead to delays in consultation and make the disease less identifiable, thus increasing the likelihood of transmission in the case of tuberculosis. To examine this hypothesis, we compared the duration of cough of the subjects before the diagnosis by Beijing and non-Beijing genotypes, because cough is the most common route of spreading the bacteria. We found that patients infected with Beijing strains had significant longer duration of cough compared to those infected with non-Beijing strains. This may partially account for why the *M. tuberculosis *Beijing genotype is more prevalent. One limitation of this observation is that the data of cough duration was based on the patients' subjective description, and this may not be very accurate. Furthermore, other socioeconomic factors may influence patient's decision for medical attention. Therefore, a larger study is needed to answer questions about the consequences of the differences in symptoms on presentation, diagnosis, and transmission of tuberculosis.

We found that there was a significant higher frequency of cavitary disease in patients infected with non-Beijing strains. However, in studies performed in Indonesia [12] and in The Netherlands [25] in which the CXR presentations of patients infected with Beijing and non-Beijing strains were compared, the authors did not find any significant difference between the two groups of patients. But in the much larger study by Drobniewski et al. [24], infection with Beijing genotype was associated more commonly with advanced radiological abnormalities, defined as multiple lung zones with fibrotic change and widespread cavitation, than was non-Beijing genotype, a result that is somewhat contrary to ours.

The plasma levels of IFN-γ and IL-18 were similar and no difference in gene expression levels of IFN-γ, IL-2 and IL-18 was observed between the Beijing and non-Beijing groups. This finding in general does not suggest that Beijing strains elicit a weaker Th1 immune response in tuberculosis patients, and differs from the observation from mouse studies that mice infected with the Beijing strains presented with reduced Th1 immune responses [13,14].

However, we found that patients infected with non-Beijing strains had significantly higher level of IL-4 gene expression in PBMCs and significantly lower IFN-γ : IL-4 cDNA copy number ratios compared to those infected with Beijing strains, suggesting a relatively enhanced Th2 immune response in non-Beijing patients. This finding to some extent agrees with a recent *in vitro *study [26] in which macrophages derived from bone marrow of mice were infected with different *M tuberculosis *genotype strains. They found that cells infected with Beijing strains induced a favorable Th1 immune response, as evidenced by the highest mRNA and protein expression of IL-1β, TNF-α, and IL-12, and a comparable IL-18 expression with other *M. tuberculosis *genotype strains. On the other hand, there were reduced Th2 responses in cells infected with Beijing strains as demonstrated by diminished IL-10 mRNA levels compared with other non-Beijing strains. This cytokine pattern is favorable to control infection. In addition, previous studies have demonstrated that increased IL-4 production is associated with advanced radiological disease [27], cavitary disease [2], and progression from latent infection to active disease [29]. In the present study, we also found that elevated IL-4 expression was related with cavitary disease. These findings suggest that IL-4 might play a pathogenic role in tuberculosis.

The limitations of this study include the small sample size which means that important differences between groups may not have been detected and some clinical data that were obtained based on patient's subjective description were fairly crude. Further studies based on a larger number of cases and with more detailed clinical and laboratory measurements would be useful to confirm and extend these findings.

## Conclusion

Our data showed that human tuberculosis caused by the *M. tuberculosis *Beijing genotype strains was associated with significantly reduced frequency of fever, night sweats, and pulmonary cavitation, suggesting that they induce less inflammatory response and tissue-damage. Th1 immune responses are similar in patients infected with Beijing and non-Beijing strains; but the increased IL-4 expression in the patients infected with non-Beijing strains suggests that non-Beijing strains activate relatively more Th2-polarized immunity compared to Beijing strains.

## Competing interests

The author(s) declare that they have no competing interests.

## Authors' contributions

YJS and NIP designed the study, drafted the manuscript, and were also involved in the subject enrolment. YJS performed the experiments and data acquisition and analysis. TKL, AKYO, BCHH were involved in the subject enrolment, TKL and AKYO also reviewed the chest X-ray films. GTS was involved in the immunological experiment design and in drafting the manuscript. All authors contributed to the final draft.

## Pre-publication history

The pre-publication history for this paper can be accessed here:


